# Estimation of Energy Expenditure Using a Patch-Type Sensor Module with an Incremental Radial Basis Function Neural Network

**DOI:** 10.3390/s16101566

**Published:** 2016-09-22

**Authors:** Meina Li, Keun-Chang Kwak, Youn Tae Kim

**Affiliations:** 1 College of Instrumentation and Electrical Engineering, Jilin University, Changchun 130061, China; 2 Department of Electronics Engineering, Chosun University, Gwangju 61452, Korea; kwak@chosun.ac.kr; 3 Department of IT Fusion Technology, Graduate School, Chosun University, Gwangju 61452, Korea

**Keywords:** energy expenditure, linguistic regression, radial basis function neural network, context-based fuzzy c-means clustering

## Abstract

Conventionally, indirect calorimetry has been used to estimate oxygen consumption in an effort to accurately measure human body energy expenditure. However, calorimetry requires the subject to wear a mask that is neither convenient nor comfortable. The purpose of our study is to develop a patch-type sensor module with an embedded incremental radial basis function neural network (RBFNN) for estimating the energy expenditure. The sensor module contains one ECG electrode and a three-axis accelerometer, and can perform real-time heart rate (HR) and movement index (MI) monitoring. The embedded incremental network includes linear regression (LR) and RBFNN based on context-based fuzzy c-means (CFCM) clustering. This incremental network is constructed by building a collection of information granules through CFCM clustering that is guided by the distribution of error of the linear part of the LR model.

## 1. Introduction

More than one-third of adults and almost 17% of youth were obese in 2009–2010 [1]. Excess nutrient and energy imbalances are considered to be a major cause of chronic diseases, such as diabetes and obesity [2], indicative of the need for a better understanding on how energy expenditure (EE) can be assessed and quantified. Body fitness and athletic performance can also be evaluated by monitoring energy expenditure and physical activities [2,3]. The conventional method to estimate energy expenditure is by using a gas system that can measure the oxygen consumption (VO_2_) and carbon dioxide (VCO_2_) in the exhaled air. The method uses a gas system and is highly accurate. However, the people tested have to wear a mask during physical activities that imposes practical limitations, since the tube needed to connect to the gas analyzer has a finite length. Furthermore, the equipment is expensive and cumbersome. Researchers have tried to develop a small sensor and algorithm to estimate the energy expenditure.

To estimate energy expenditure, two commonly used methods include the monitoring of heart rate (HR) and sensing of movements. Spurr et al. developed the FlexHR method that uses the resting metabolic rate (RMR) and exercise activity to estimate energy expenditure [4]. The FlexHR point is the highest HR in the RMR compared to the lowest HR during exercise. If the HR is lower than the FlexHR, then energy expenditure is estimated based on the RMR, whereas if it is higher, then the estimation is based on the linear relationship between energy expenditure and VO_2_. This method has been extensively applied in research lately. However, the accuracy of the elicited results requires them to be fitted to a linear regression model to develop the estimation formula. Another method uses movement sensors that capture the motion of the trunk [5,6], chest [7,8], hip [9], and waist [10], for estimating the daily energy expenditure [11]. Movement sensors contain a tri-axial accelerometer that measures acceleration in the vertical, horizontal, and mediolateral planes. It can monitor various physiological and movement parameters that can be used in conjunction with the subject’s gender, age, height, and weight, to estimate the energy expenditure with a generalized algorithm [12,13,14]. Another use of the movement sensor is that it can indicate the exercise intensities, which can help to differentiate the increases in HR due to activity or due to emotional stress [15]. To increase the accuracy of this method, the number of sensors placed on the human body, and at different body postures, must be increased in order to obtain various parameters [16]. An alternative method employs a combination of the HR and motion sensors to estimate energy expenditure [17,18,19]. Here, motion sensors are used to exclude any increases in the HR for reasons other than exercise [20]. The effectiveness of this combined-sensor method remains unclear because it may underestimate high-intensity activities, while overestimating low-intensity ones [21]. Its accuracy has been evaluated through laboratory experiments, but a free-living environment is more complex than that found in a laboratory in terms of parameters, such as the stride length and speed.

Another approach adopted to improve the accuracy is to use machine learning. Dong [22] used wearable multisensors to extract features of different activities for the construction of the artificial neural network and linear regression. In [23], the authors presented three regression techniques, namely, least squares regression, Bayesian linear regression, and Gaussian process regression, with the data from treadmill walking using hip-worn inertial sensors. The results show that the nonlinear methods have better prediction accuracy. Wang [24] presents a portable-accelerometer and electrocardiogram sensor system with a machine learning-based model for energy expenditure. Lin [25] estimated the daily energy expenditure using a wearable sensor module with a neural network based on an activity classification algorithm. They employed forward and backward search strategies for feature selection. Radial basis function neural network (RBFNN) and generalized regression neural network (GRNN) models were employed as energy expenditure estimation models. The existing literature on the aforementioned lists techniques that have been performed based on how well the regression or neural networks are known from the numerical data.

Energy expenditure estimation techniques that use heart rate, movement, and combined methods have been described in the literature. These methods are based on built linear models. However, because of the various activities involved in daily life, the data set cannot always be kept online, which may reduce the accuracy of estimation in these models. Therefore, in this study, we propose a patch-type sensor with an embedded network architecture that can integrate the nonlinear components as input variables for energy expenditure estimation. We call this embedded network architecture the incremental RBFNN.

**Figure 1:**
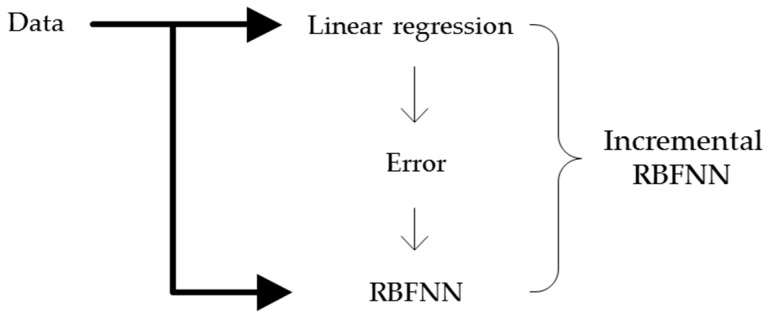
General flow of the development of the incremental RBFNN.

The structure of the incremental RBFNN comprises two parts, as shown in Figure 1. The first layer is the construction of a standard regression model, which could be treated as the preliminary construct capturing the linear part of the data, thereby forming the backbone of the entire construct. Next, all modeling discrepancies are compensated by a collection of receptive fields that become attached to the regions of the input space where the error is localized. The receptive fields are in the subspaces of the original input space rather than in the entire input space. The incremental RBFNN is reconstructed by building a collection of information granules formed by using the context-based fuzzy c-means (CFCM), which is guided by the distribution of error of the linear part of the model. The so-called CFCM is the input space and is conditioned based on linguistic landmarks. We determine the optimal context of the input variables by successive trials and errors in order to realize a high accuracy output. Simply, the principle of the designed architecture can be summarized as follows: (a) adopt the construct of a linear regression as a first-principle, “global” model; and then (b) refine it through a series of “local” receptive fields that capture the remaining and more “localized” nonlinearities of the system.

The main scope of this study is to estimate the EE of walking and running which are the most common activities for adults in modern lifestyles. The rest of this paper is organized as follows: in Section 2, we illustrate the system architecture that includes the entire generation of the system and the structure of the patch-type sensor. The embedded incremental RBFNN is described in detail in Section 3. Section 4 then provides the experimental design and results in accordance to a two-part protocol. First, the experiment is conducted using a submaximal treadmill protocol in a laboratory. Then, we instructed the participants to walk and run on a school playground as naturally as possible. Finally, conclusions are summarized in Section 5.

## 2. System Architecture

### 2.1. General System Description

The function of the entire system is wireless monitoring of the energy expenditure of the participants using a cheap and lightweight sensor. The users can be monitored at any time during the day and upon engagement in different physical activities. The system includes one inverse triangle patch-type sensor and a portable computer to display the heart rate and movement index (MI). The sensor node is small in size (6 × 9 cm^2^) and lightweight (41 g) and can be patched on the chest of the user for collecting physiological data. The physiological data include HR, MI, humidity, and temperature. The sensor has the ZigBee RF module that can wirelessly transmit the information to a personal computer. The analysis software is embedded in the portable computer. The physician could check the physiological condition of the user using the portable computer.

### 2.2. Sensor Module

**Figure 2:**
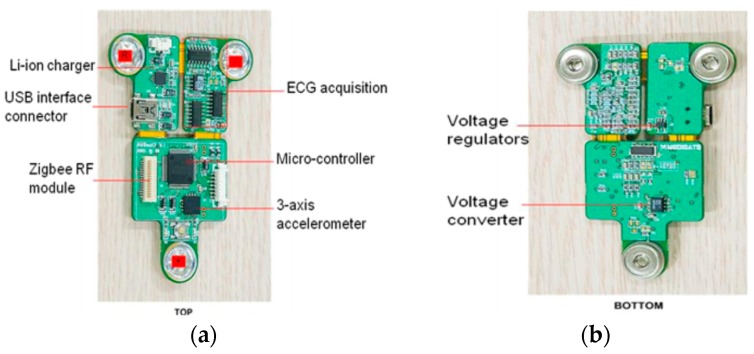
(**a**) Top and (**b**) bottom views of the sensor node.

The sensor board consisted of a three-axis accelerometer, three ECG electrodes, a voltage converter, and a Li-ion charger. Two quasi-triangular flexible PCB boards are combined in the sensor, as shown in Figure 2. The MI is the summation of the motion indices along all the three axes x, y, and z. It can detect the movement acceleration signal in the range of −6 g to 6 g. The agility was examined using physical training protocols, such as the zigzag run, a 20 m shuttle run, the Burpee test, and a side-step test. The movement detection was strongly correlated to conventional agility tests (R^2^ = 0.80–0.91). The results verified its applicability to the evaluation of the exercise state of the users. The HR detection was designed based on the use of a bandpass filter for R-wave detection. Motion artifacts were incurred and led to an error rate that was less than 2% with a commercial stress ECG monitor (CASE System, GE Medical, Westborough, MA, USA). The sensor node included 3.3 V Li-ion rechargeable batteries with a continuous operation period of two hours. The distances for transmission can achieve more than 400 m in an open field using the ZigBee telecommunication. The technical details of the sensor design can be found in a previously published study [26].

## 3. Incremental RBFNN

**Figure 3:**
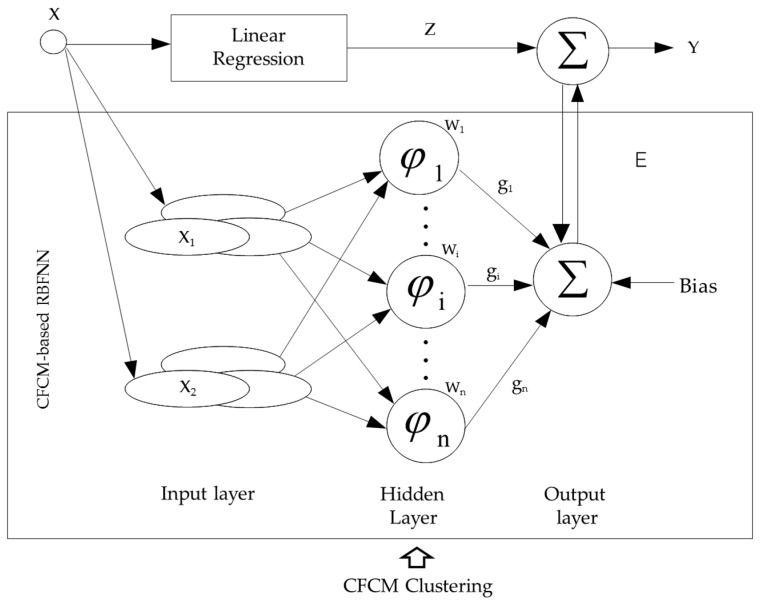
Overall flow of processing realized in the design of incremental RBFNN.

The overall design process of the incremental RBFNN is shown in Figure 3. The development of the incremental RBFNN consists of two phases. First, we design the linear regression (LR) phase, which is treated as the preliminary global model representing the linear part of the data. Next, the modeling error obtained by the LR is compensated by the local RBFNN. In the design of RBFNN, the hidden layer is constructed using fuzzy granulation realized via CFCM clustering.

For simplicity, we assume that the incremental RBFNN under consideration has two inputs, $x_{1}$ and $x_{2}$. We firstly design the LR model in the input-output space. On the basis of the original data set and error obtained by the LR model, the nonlinear data sets are formed in a collection of input-error pairs. The contexts are produced from the input-error pairs and are characterized by the triangular membership functions.

### 3.1. RBFNN

The RBFNN is attractive in that it can be used for functional approximations, prediction, interpolation, and nonlinear modeling [27], which makes it useful in many applications. The RBFNN has a three-layer structure: an input layer that feeds feature vectors into the network, a hidden layer that calculates the outcome of radial basis functions, and an output layer that calculates a linear combination of basic functions. The receptive fields $w_{i}$ of the *i*th hidden unit in the hidden layer are calculated as follows:

(1) $$w_{i}=\phi_{i}\left(\left\|x-v_{i}\right\|\right)=\exp\left[-\frac{{\left(x-v_{i}\right)}^{2}}{2\sigma_{i}^{2}}\right]$$

where x is a multi-dimensional input vector, and $\phi_{i}$ is the *i*th radial basis function in the hidden layer. Additionally, $v_{i}$ is the center of the Gaussian basis function associated with *i*th hidden unit, and $\sigma_{i}$ is a width parameter of *i*th hidden unit. The output of the hidden units is normalized between 0 and 1. The output of RBFNN is determined as follows:

(2) $$E=\sum_{i=1}^{n}g_{i}w_{i}$$

where $g_{i}$ is the weight from the hidden unit to output E. From the above equation, we can determine on whether the performance is highly depended on the receptive fields and on the connections of the neuron in the output layer of network. The RBFNN is a two-layer feed-forward network. The training process of the RBFNN is divided into two stages: (a) first, the centers from the input to the hidden layer are determined; and (b) the weights are then determined from the hidden to the output layer. Once we have formed the receptive field, the optimization of the weights of the neuron in the output layer becomes straightforward [28,29]. Since the centers and widths are fixed after they are chosen, the RBFNN often results in an unsatisfactory performance when the input patterns are not particularly clustered. Therefore, the CFCM clustering is used in conjunction with the RBFNN to determine good locations for the radial basis functions.

### 3.2. Local RBFNN Based on CFCM Clustering

CFCM clustering partitions a collection of input vectors into several fuzzy groups and estimates a cluster center in each context, such that a cost function of dissimilarity measure is minimized. In what follows, we shall briefly describe the CFCM clustering algorithm. The given data belong to corresponding membership values. The partition matrices were induced by the *l*th context and are denoted and defined as follows

(3) $$U=\left\{u_{\mathrm{ik}}\in \left[0,1\right]|\sum_{i=1}^{c}u_{\mathrm{ik}}=w_{\mathrm{lk}}\;\forall k\;\mathrm{and}\;0<\sum_{k=1}^{N}u_{\mathrm{ik}}<N\;\forall i\right\}$$

where $w_{\mathrm{lk}}$ is a membership value of the *k*th data point implied by the *l*th context. The underlying objective function can be expressed in the standard format as follows:

(4) $$Q=\sum_{i=1}^{c}\sum_{k=1}^{N}u_{\mathrm{ik}}^{m}{\left(x_{k}-v_{i}\right)}^{2}$$

where m is any real number greater than 1, u_ik_ is the degree of membership of $x_{k}$ in the *k*th cluster, and ‖∙‖ is a distance function between any measured data and the *i*th center cluster. The minimization of the objective function is realized by iteratively updating the values of the partition matrix and the prototypes, as shown above. The update of the partition matrix u_ik_ and the cluster center $v_{i}$ is computed as follows

(5) $$u_{\mathrm{ik}}=\frac{w_{\mathrm{lk}}}{\sum_{j=1}^{c}{\left(\frac{\left\|x_{k}-v_{i}\right\|}{\left\|x_{k}-v_{j}\right\|}\right)}^{\frac{2}{m-1}}}$$

where u_ik_ pertains to the partition matrix induced by the *l*th context. The prototypes produced for the Euclidean distance are calculated in accordance to

(6) $$v_{i}=\frac{\sum_{k=1}^{N}u_{\mathrm{ik}}^{m}x_{k}}{\sum_{k=1}^{N}u_{\mathrm{ik}}^{m}}$$

The cluster centers of Equation (6) estimated by CFCM clustering are used in the hidden layer of RBFNN described in Section 3.1.

The main design procedure of incremental RBFNN consists of the following steps:

[Step 1]: Perform LR modeling from the original input-output data points. Here, the residuals obtained by LR are used as output variables in the design of the local RBFNN, based on CFCM clustering.
[Step 2]: Generate contexts in the residual (output) space. These contexts are produced through a uniform distribution, while the contexts are characterized by triangular membership functions with a half overlap between neighboring contexts. The cluster centers in each context are then estimated using CFCM clustering. Here, the number of the final clusters is c × p, where p is the number of contexts, and c is the number of clusters in each context.
[Step 3]: Design the RBFNN based on CFCM clustering. Here, the number of nodes in the hidden layer is equal to that of the final cluster. The weights are obtained using least squares estimation (LSE) in one-pass, without back-propagation (BP).
[Step 4]: Combine the outputs of LR and RBFNN as follows
Y = z + E
(7)
  where Y is the final output of the incremental RBFNN, as shown in Figure 3. The z and E are the outputs of the LR and the local RBFNN, respectively. Therefore, the modeling error based on LR as the global model is compensated by the local RBFNN, based on specialized CFCM clustering using the concept of information granules.

## 4. Experimental Design and Results

To evaluate the function of incremental RBFNN and realize the final goal applied under free-living conditions, the design of the experiment consisted of two parts, namely, a laboratory, and a field test.

### 4.1. Laboratory-Based Experiment

**Figure 4:**
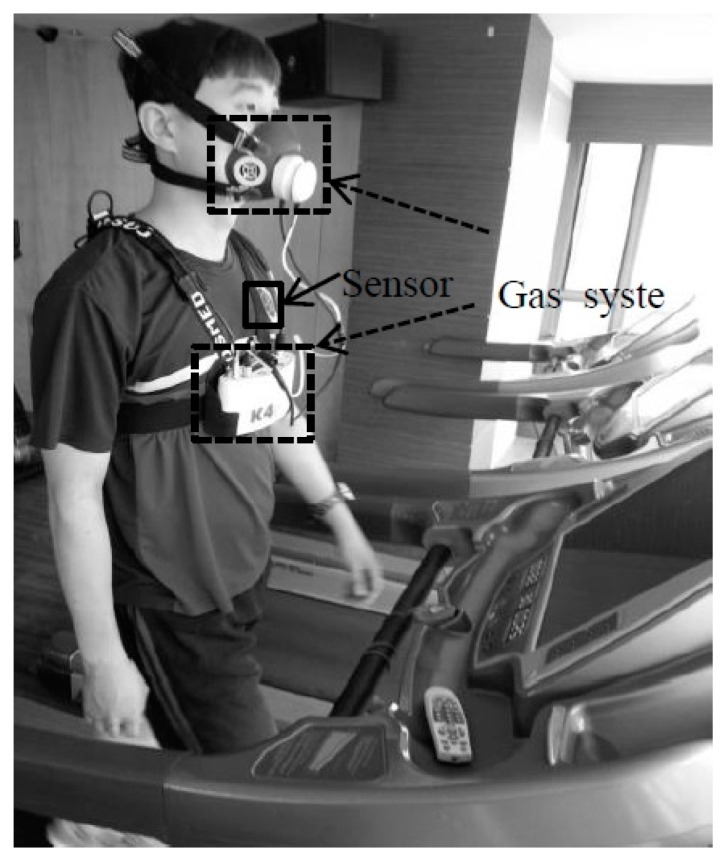
Experimental setup on the treadmill.

The participants were tested on a treadmill using the submaximal Bruce protocols in the laboratory, as shown in Figure 4. The treadmill’s initial velocity was set at 9.72 m∙s^−1^, and with a 10% gradient. The incline of the treadmill was increased by 2% at three-minute intervals. The participants walked and ran until they felt exhausted. For men, the test time lasted approximately 12 min, whereas for women the test time lasted approximately 9–10 min. The HR and MI were recorded in real-time by the patch-type sensor. The VO_2_ and EE were measured by the gas system. The physical characteristics of the subjects are listed in Table 1.

HR and MI are the two major factors used to estimate EE. Thus, they were considered as two inputs to the incremental RBFNN. The total number of data pairs is 252. In order to solve the data scarcity problem, we divided the data set into training and test datasets as a commonly used solution. The training datasets were randomly selected from approximately 60% of the entire dataset, and the testing dataset from the remaining 40%. The data were normalized between 0 and 1. Ten iterations were used for all experiments. The training data were used for the construction of the incremental RBFNN. However, the testing data were used for the verification of the network. Thus, the resultant network is not biased towards the training dataset, and it is likely to have a better generalization capacity to new data. The nonlinear data we called “error” can be calculated using the RMSE Euclidean value, as shown in Equation (8).

(8) $$\mathrm{RMSE}=\sqrt{\frac{\sum_{K=1}^{N}{\left(EE_{k}-EE_{k}^{\prime }\right)}^{2}}{N}}$$

where N is the length of the training or testing datasets, *EE_k_* is the estimated EE, and *EE_k_’* is the measured EE.

**Figure 5:**
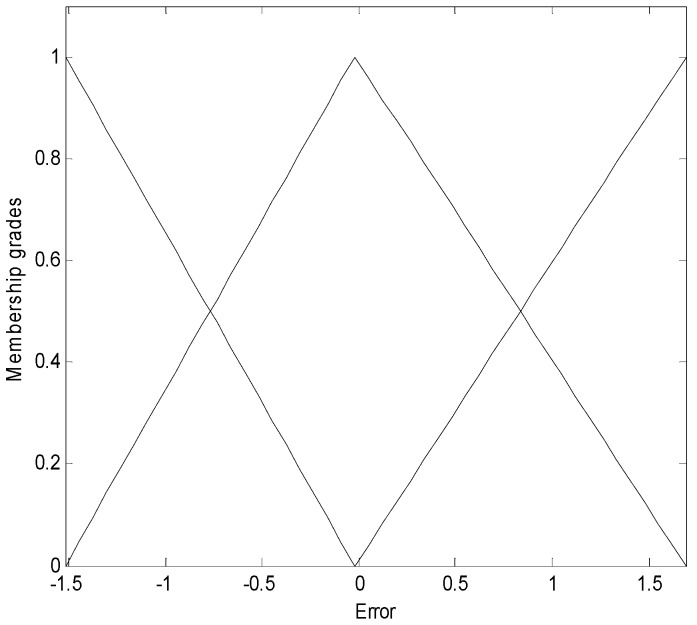
Linguistic contexts produced by the error distribution for treadmill data (p = 3).

The error values constituted the context dataset. These contexts are generated through the triangular membership functions, and are equally spaced along the domain of an output variable. The membership matrix is initialized between 0 and 1, as shown in Figure 5 (context p = 3).

**Figure 6:**
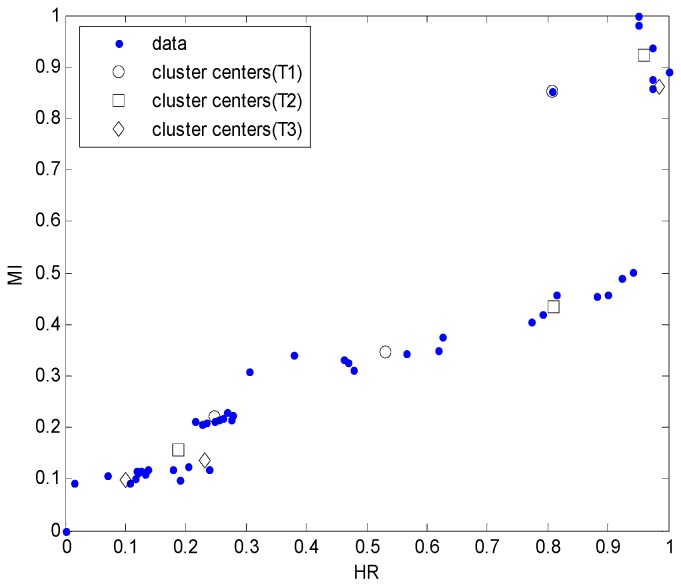
Estimation of cluster centers in each context (p = 3).

The centers can be generated by fuzzy c-means clustering based on each of the contexts. Given “p” contexts and “c” centers per context, c × p clusters can be obtained. Figure 6 shows the cluster centers generated by the three contexts and the three clusters. The cluster centers can be the same as those of the RBFN hidden layer. Therefore, the RBFN model can be constructed as follows: HR and MI are used as the inputs of the input layer, nine units as the hidden layer—based on the number of contexts—and the clusters, and the EE as the output layer.

The estimation of the performance was evaluated by the RMSE as the number of context, and the cluster increased from three to six and from two to six, respectively. Table 2 shows the RMSE results for the training and testing data. The result elicited from the best fitting model is shown in bold (p = 3, c = 3). The comparison of the RMSE with previous work is shown in Table 3. The RBFNN we used was a 2–10–1 network, employed a BP algorithm, and 1000 epochs with a learning rate of 0.01. The linguistic model (LM) we used consisted of three contexts and three clusters which were determined by trial and error. The CFCM-RBFNN used the hidden layer that increased from 3 to 20 in the experimental design.

### 4.2. Field Test

**Figure 7:**
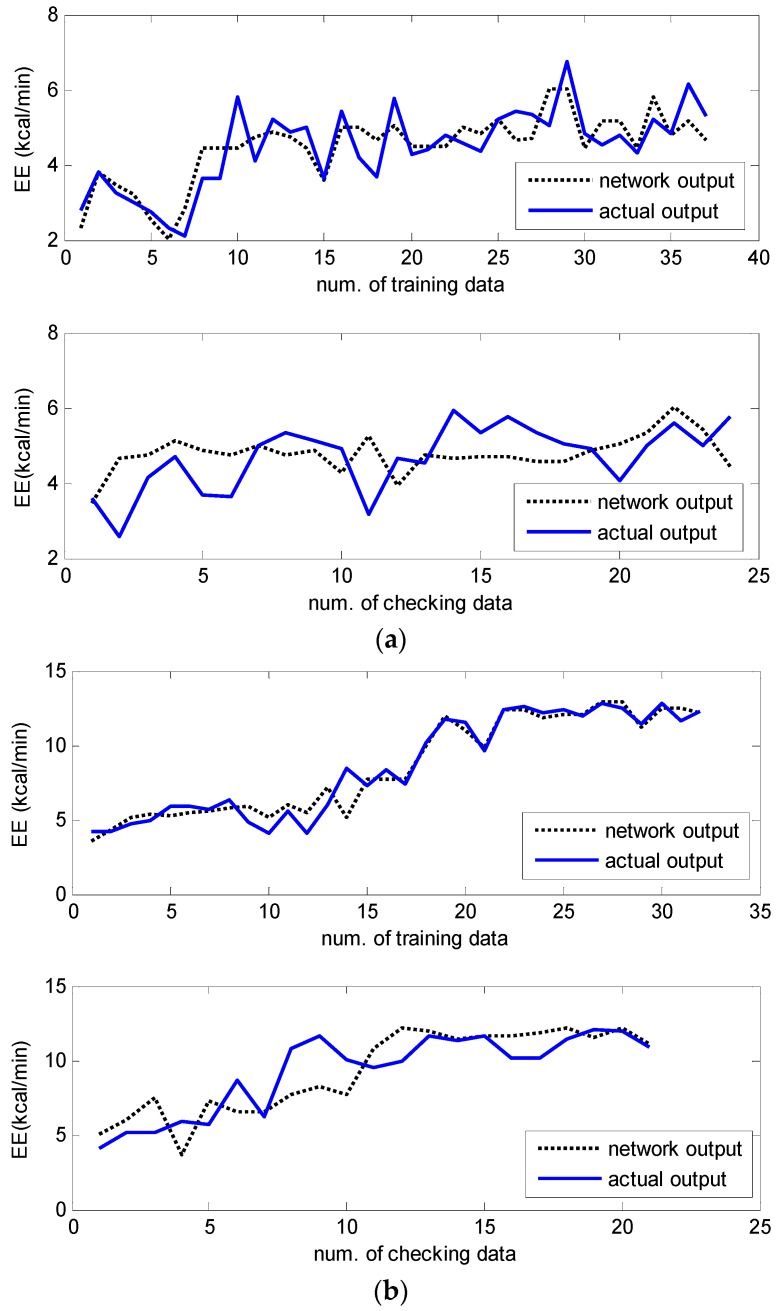

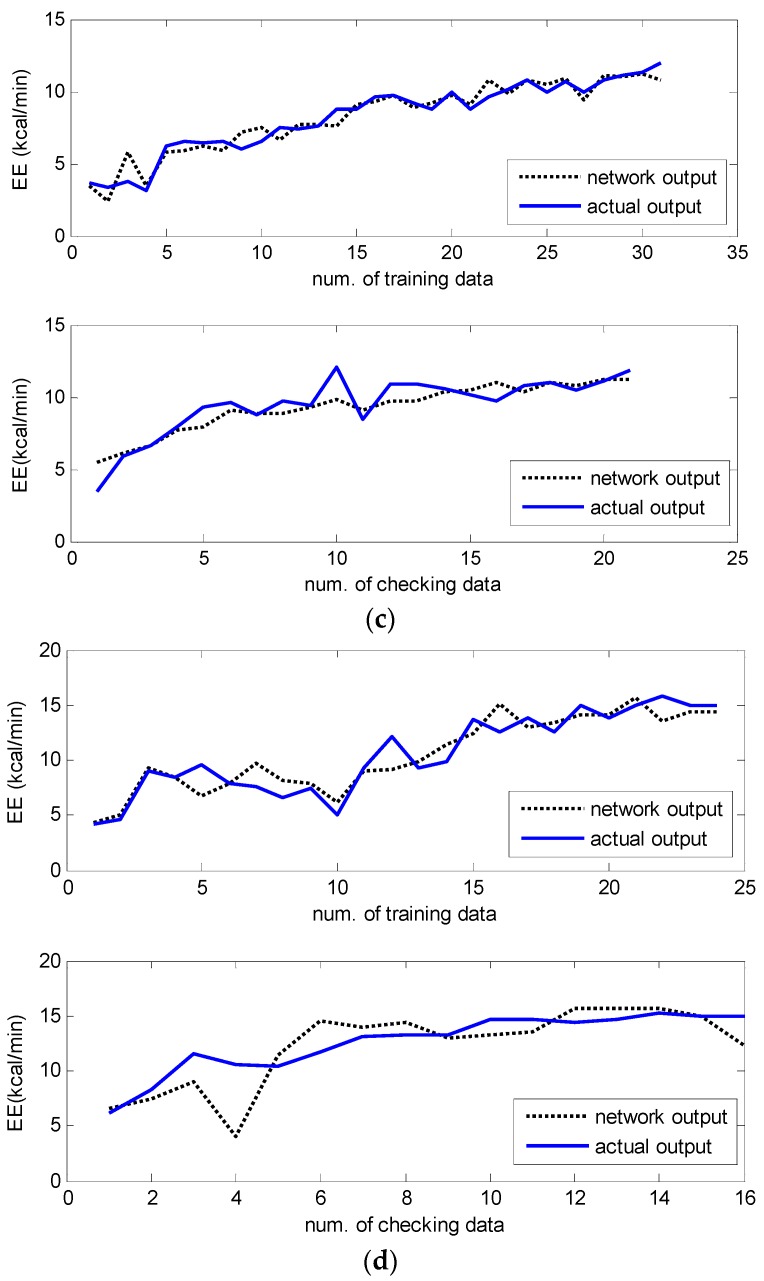
(**a**–**d**) Prediction performance of energy expenditure by the incremental RBFNN. (**a**) Normal walking in school playground; (**b**) Brisk walking in school playground; (**c**) Slow running in school playground; (**d**) Jogging in school playground.

The final goal is to realize the accurate estimation of EE under free-living conditions. After the laboratory tests, all of the participants were encouraged to complete four exercise tests in the open field. The first was comfortable walking, the second jogging, the third was a quick walk, and the last was slow running. Each test course was performed in approximately two minutes in an oval track field. The experimental procedure was designed to progress as naturally as possible. The structure of the RBFNN thus constituted the reference basis of the experimental laboratory test. The data obtained for walking, jogging, fast walking, and slow running, respectively consisted of 157, 60, 63, and 99, sample pairs. The prediction performance is shown in Figure 7. As indicated in the Figure 7, the experimental results revealed that the proposed network showed good prediction and generalization performance in all cases of normal walking, brisk walking, slow running, and jogging, for the training and testing datasets, respectively. Table 4 lists a comparison of the RMSE data collected using the RBFNN and LM methods.

## 5. Conclusions

This study has shown that the incremental RBFNN is effective for estimating the EE. The significant advantage of this model is that it starts from the linear regression model and then uses a refined version of this regression model by adding granular patches. This leads to a final model that is quite different compared to the preliminary version of the linear model. The other important design is related to the application of the CFCM clustering for the calculation of the center value, which is a process that can reduce the iteration times and optimize the network structure. The output space can be optimized by the context number and the number of clusters. The experimental results have been compared with the LM, RBFN, and CFCM-RBFNN, which indicate that the sensor module that is based on the incremental RBFNN, has the ability to estimate the EE during walking and running both in laboratory and free-living settings. Moreover, in future work, the same approach of the incremental modeling could be explored in other domains, such as pattern recognition and classification, which could be especially useful for application to the e-health monitoring field.

## Figures and Tables

**Table 1:** Physical characteristics of the participating subjects (*N* = 30).

Variable	Men (*N* = 17)		Women (*N* = 13)	
	Mean	Range	Mean	Range
Age, year	26 ± 2.1	24–27	25.8 ± 3.2	23–28
Height, cm	169 ± 6.7	167–180	162.1 ± 6.3	155–165
Weight, kg	65.2 ± 9.6	59–70	52.1 ± 9.4	48–57
BMI, kg·m^−2^	22.8 ± 7.1	20–23	19.8 ± 4.1	18.6–21.7

**Table 2:** RMSE in the incremental RBFNN model for the training and testing datasets.

**Treadmill Data (Training Data)**		**Number of Contexts**			
		**3**	**4**	**5**	**6**
Number of clusters per context	2	0.5237	0.5089	0.4798	0.4557
	3	0.4722	0.4436	0.4182	0.3834
	4	0.4367	0.3832	0.3605	0.3276
	5	0.3935	0.3659	0.3050	0.2425
	6	0.3691	0.3245	0.2528	0.1821
**Treadmill Data (Testing Data)**		**Number of Contexts**			
		**3**	**4**	**5**	**6**
Number of clusters per context	2	0.6913	0.7506	0.7375	0.7563
	3	0.6627	0.7109	0.7197	0.8622
	4	0.6944	0.7903	0.7819	0.8641
	5	0.7321	0.7162	3.6389	10.938
	6	1.0113	1.1930	99.955	261.54

**Table 3:** Comparison of RMSE values for treadmill data.

	Trn_RMSE	Txt_RMSE
RBFNN [22]	0.73	0.97
LM (p = 3, c = 3)	0.65	0.95
RBFNN-CFCM [18]	0.64	0.95
Incremental RBFNN	0.47	0.66

**Table 4:** Comparison of RMSE values for field exercise data.

Activity	Method	Trn_RMSE	Txt_RMSE
Walking	LM	0.75	1.06
	Incremental RBFNN	0.60	0.95
Brisk walking	LM	1.47	1.82
	Incremental RBFNN	0.96	1.68
Slow running	LM	0.79	1.44
	Incremental RBFNN	0.61	1.07
Jogging	LM	2.0	2.58
	Incremental RBFNN	1.32	2.45
